# Evolutionary and Developmental Modules

**DOI:** 10.3389/fncom.2013.00061

**Published:** 2013-05-17

**Authors:** Francesco Lacquaniti, Yuri P. Ivanenko, Andrea d’Avella, Karl E. Zelik, Myrka Zago

**Affiliations:** ^1^Department of Systems Medicine, University of Rome Tor VergataRome, Italy; ^2^Centre of Space Bio-Medicine, University of Rome Tor VergataRome, Italy; ^3^Laboratory of Neuromotor Physiology, IRCCS Santa Lucia FoundationRome, Italy

**Keywords:** activation pattern, CPG, gene expression, interneurons, locomotion

## Abstract

The identification of biological modules at the systems level often follows top-down decomposition of a task goal, or bottom-up decomposition of multidimensional data arrays into basic elements or patterns representing shared features. These approaches traditionally have been applied to mature, fully developed systems. Here we review some results from two other perspectives on modularity, namely the developmental and evolutionary perspective. There is growing evidence that modular units of development were highly preserved and recombined during evolution. We first consider a few examples of modules well identifiable from morphology. Next we consider the more difficult issue of identifying functional developmental modules. We dwell especially on modular control of locomotion to argue that the building blocks used to construct different locomotor behaviors are similar across several animal species, presumably related to ancestral neural networks of command. A recurrent theme from comparative studies is that the developmental addition of new premotor modules underlies the postnatal acquisition and refinement of several different motor behaviors in vertebrates.

## Introduction

Modules are easily defined in human-engineered product design. The final product is split up into components (modules) that are designed, manufactured, and tested independently. The modules can be reused in different systems that perform different tasks. Modular industrial design tends to reduce manufacturing complexity and costs. Parsimony, reduction of complexity, and evolutionary costs presumably also underlie modular solutions in biological systems, but biological modules are not always easily identifiable. We will return later to the issue of cost factors possibly underlying the evolution of modules. Here we first address the issue of the identification of biological modules.

According to Fodor (1983), modular systems are decomposable in a set of independent processes. However, the degree of independence among the basic components is not fixed. According to Simon (1969), the interactions among components are negligible in comparison with those within components only in fully decomposable systems. Instead, nearly decomposable systems are those in which the interactions among components are weak but not negligible. Finally, un-decomposable systems are those in which the interactions among components are as strong as those within components.

A biological module could be defined on the basis of structural (morphological), functional, or developmental elements. A structural module is defined by a set of spatially defined, inter-connected elements. A functional module is a discrete entity whose function is separable from that of other modules (Hartwell et al., 1999). A developmental module is a component of a developing organism (e.g., an embryo) that is semi-autonomous relative to pattern formation and differentiation, or relative to a signaling cascade (Schlosser and Wagner, 2004).

Spatially bounded structures (e.g., the ribosomes) are the most clearly defined modules at a subcellular level. At a functional level, the ensemble of cellular proteins (proteome) can be partitioned into a limited set of complexes, which differentially combine to support diverse functions (Ravasz et al., 2002; Gavin et al., 2006). The identification of biological modules at the systems level is even more challenging. Top-down decomposition may help recognizing a modular structure. A complex event (such as the goal of a motor task) can be detailed by decomposing it in several more basic informational events. If the system being analyzed is structured as a hierarchy, decomposition can be performed recursively (iteratively), each stage leading to finer and finer basic components (Palmer and Kimchi, 1986). Successive decompositions remove some of the complexity that is inherent in higher levels of description, but they raise the issue of when to stop. Ideally, one would like to stop when a stage of primitive informational events has been reached. One could take as primitive some computationally plausible set of operations that is sufficient to perform the task. This approach has been used in the study of vision, for instance (Palmer, 1999). Thus, the task of visual object identification is decomposed in the distinct operations involved in image-based, surface-based, object-based, and category-based processing.

Modules may also be identified using bottom-up decomposition, instead of top-down decomposition. Starting from a multidimensional array (e.g., electromyographic EMG activity), we aim at identifying basic components that represent some common features, which are shared by a number of the original data (for instance, the output variables in a motor task, Lacquaniti et al., 2012b; d’Avella and Lacquaniti, 2013). Here the basic components represent building blocks that are used to construct a given behavior; therefore, they can be considered as primitives in a computational sense (Flash and Hochner, 2005; Bizzi et al., 2008; Giszter et al., 2010).

Top-down and bottom-up approaches can be merged together using hierarchical multiple layers of representation. For example, hand action representations have been modeled as involving a temporal postural synergy (one kind of motor module) only if the ancestors of the synergy in the tree-structured organization are themselves involved in the action representation (Tessitore et al., 2013). The advantage of combining the two approaches consists in the possibility of validating an *a priori* hypothesis about the structure of the task implied in top-down decomposition with the *post hoc* identification of basic components derived from bottom-up decomposition.

## Evolutionary and Developmental Perspectives

The approaches described above have been applied primarily to mature, fully developed systems. A different perspective on modularity is provided by comparative developmental and evolutionary studies (e.g., Gilbert et al., 1996; Wagner and Altenberg, 1996; Winther, 2001; Schlosser and Wagner, 2004; Flash and Hochner, 2005; Cheung, 2007; Gerhart and Kirschner, 2007; Raff, 2007; Giszter et al., 2010). Evolutionary developmental biology (often referred to as Evo-Devo) aims at understanding how evolutionary trajectories (phylogeny) are constrained by developmental rules (ontogeny), and how developmental rules themselves evolve. Thus, processes evolve to produce new patterns of development, new gene regulation, new morphologies, and new behaviors (Raff, 2007).

There is now much evidence that modular units of development were highly preserved and recombined during evolution. It has been argued that biological modularity is the result of evolution and facilitates evolution (Schlosser and Wagner, 2004). Evolvability, that is, the capacity of a system for adaptive evolution, is positively affected by modularity when the developmental modules of an organism match the modularity of specific adaptive functions (Kirschner and Gerhart, 1998; Schlosser and Wagner, 2004).

There are several applications of the concept of modularity from an evolutionary developmental perspective. We first consider a few examples of anatomical modules, because these are relatively well identifiable and represent a model for the more difficult identification of modules in motor control. During development, anatomical modules form integrated series of relatively distinct, autonomous components that help partitioning different parts of the embryo, reducing the effects of any changes on the organism as a whole (Reno et al., 2008). The developmental modules have a genetically discrete organization, resulting in identifiable domains within the developing organism, and these modules undergo evolution (Raff, 2007). The most basic modular gene expression networks provide the crucial regulation of body-plan development at the phylum level of the animal kingdom (Raff, 2007).

A striking example of genetically controlled modularity has been demonstrated by Halder et al. (1995). They obtained ectopic eyes on the wings, legs, and antennae of *Drosophila* by misexpression of the cDNA “eyeless” gene. (The products of “eyeless” and twin of “eyeless” gene are homologs of the vertebrate Pax6.) The ectopic eyes consisted of complete eye structures and were responsive to light, indicating that “eyeless” is the control master for the genesis of the fly eye.

Studies of fossils of Placodermi (prehistoric fishes) showed separate evolution of jaws and teeth, through distinct developmental modules, topographically related but each independently genetically regulated (Smith and Johanson, 2003; Rücklin et al., 2012). Comparative embryological studies showed that the transcription factor Satb2 specifies a developmental module within the mandibular jaw (Fish et al., 2011). Satb2 is expressed in the mesenchyme of the jaw primordia that gives rise to distal elements of both the upper and lower jaws.

The development of the limbs and their evolutionary adaptation to locomotion and other functions provide another important example of modularity. Limb development begins in the embryological limb field, followed by the ectoderm bulging out as the limb bud. Certain homeobox (Hox) genes act as selector genes with spatially regulated expression patterns that specify differential growth in distinct modules. Hox genes are widely shared across the animal kingdom, from insects to reptiles and mammals. In general, these genes are linearly ordered in a sequence which maps into the spatial order and timing of development of different body regions. Duplication of Hox genes can produce new body segments, and this mechanism probably played an important role in the evolution of segmented animals. In particular HoxA and HoxD specify segment identity along the limb axis.

It is well known that tetrapod limbs have evolved from fish fins. Teleosts are ray-fin fishes that diverged from the lobe-fin ancestors of tetrapods more than 400 millions of years ago. The fin buds of the living zebrafish (a teleost) exhibit the early phases of Hoxd13 expression that are observed in tetrapod limb buds, despite the fact that the appendages of zebrafish have no skeletal homologs in tetrapods (Raff, 2007). Zebrafish shows walking-like movements of the pectoral fins during slow swimming (Thorsen et al., 2004). Late-phase HoxD expression pattern was present in primitive bony fish and was lost together during evolution with the posterior portion of the ancestral fin in teleosts. It was retained, however, in sarcopterygians like *Tiktaalik*, and co-opted into the tetrapod limb (Raff, 2007). *Tiktaalik* was an extinct fish (late Devonian) with limb-like fins that probably allowed it “walking” on land.

Hoxa and Hoxd gene expression patterns also demarcate boundaries of several developmental domains in the limbs of birds and mammals. Thus, the five HoxD genes expressed in distal limb buds regulate the formation of the five digits in living tetrapods. In particular, anthropoids (humans, apes, new world, and old word monkeys) exhibit differential adaptations in forearm and digital skeletal proportions to the specific locomotor modes. It has been argued that Hox-defined developmental modules may have served as evolutionary modules during hand evolution in anthropoids (Reno et al., 2008). On the basis of forearm and digital morphometric data in several anthropoid species and of correlations with the spatial patterns of Hoxd expression territories during bone growth, Reno et al. (2008) postulated the existence of at least two developmentally independent growth modules: (1) a posterior digit module which includes the distal radius, posterior digit metacarpals, and phalanges of the long digits, and (2) a distal thumb module. The growth of the posterior digit module is possibly regulated by Hoxd11, and the thumb module by Hoxa13 and Hoxd13. Thus, the posterior digit elongation observed in ateline and colobine (a new world and old word monkey, respectively) could have been achieved through an up-regulation of Hoxd11 expression (Reno et al., 2008). An elongated thumb, on the other hand, may interfere with brachiation; thus, thumb reduction in atelines and colobines may have been an adaptation to brachiation, and may have resulted from the modulation of Hoxa13/Hoxd13 expression. The length of the radius and posterior digits in humans is short relative to that of other higher primates. Instead, humans have very long thumb phalanges. This pattern could be achieved by the combination of up-regulation of the targets of Hoxd13 and/or Hoxa13 expression, and down-regulation of Hoxd11 targets (Reno et al., 2008).

## Spinal Circuits for the Control of Locomotion

The identification of functional modules in motor control is more difficult than that of anatomical modules, but similar principles of evolutionary conservation probably apply. In particular, there is growing evidence that the building blocks used to construct locomotion are similar across several animal species, presumably related to ancestral neural networks of command. In all vertebrates, spinal neuronal networks termed Central Pattern Generators (CPGs) generate the basic rhythms and patterns of motor neurons (MNs) activation for locomotion (Grillner, 2006). CPGs are controlled by descending supraspinal inputs (including locomotor command regions in the brainstem), as well as by sensory inputs. Comparative studies in vertebrates using genetic and electrophysiological tools have consistently shown that there are several common principles in the organization and regulation of CPGs (Goulding, 2009; Kiehn, 2011). In particular, the core premotor components of locomotor circuitry mainly derive from a set of embryonic interneurons which are remarkably conserved across different species (Goulding, 2009). Grillner (2011) suggested that the neural control system for locomotion can be traced back to the lamprey, a jawless fish-like vertebrate, which appeared about 560 million years ago, before any legged animal had evolved yet. Notice that not only the spinal CPG modules, but also some supraspinal centers which contribute importantly to locomotor control, such as the basal ganglia, have been conserved throughout vertebrate phylogeny starting with the lamprey (Grillner et al., 2013).

In addition to the segmentally organized MNs that innervate adjacent myotomes, there are a few basic classes of neurons in the vertebrate locomotor CPG with established homologies across different aquatic (lamprey, *Xenopus*, zebrafish) and terrestrial (mouse) species (Grillner, 2006; Goulding, 2009; Kiehn, 2011). (1) Glycinergic inhibitory commissural interneurons project to the opposite side of the spinal cord, and provide the mid-cycle inhibition underlying left-right alternation: the muscles on each side of the body must contract out of phase with those on the opposite side. These interneurons are termed inhibitory CINs in the lamprey and *Xenopus*, CoSA neurons in the zebrafish, and V0_D_ neurons in the mouse. (2) Ipsilaterally projecting inhibitory interneurons provide inhibition to MNs and to commissural interneurons, and can regulate the speed of locomotion. They are termed L-interneurons in the lamprey, CiA neurons in the zebrafish, and V1 neurons in the mouse. The V1 class includes Renshaw cells and Ia-inhibitory interneurons. (3) Glutamatergic excitatory interneurons project to the other CPG cell types. A number of these interneurons provide rhythmic drive to MNs and other CPG neurons. They are termed EIN in the lamprey, CiD neurons in the zebrafish, and V2a neurons in the mouse.

The close correspondence of several classes of spinal CPG neurons across aquatic and terrestrial vertebrates suggests that the corresponding neuronal modules may have been evolutionarily conserved between the swimming and walking CPG. This close phylogenetic relationship is especially evident in the embryonic spinal cord (Goulding, 2009; Kiehn, 2011). Nevertheless, the full neural circuitry required for legged-locomotion is more complex than that required for swimming, presumably in relation to the different biomechanics of these modes of locomotion (Goulding, 2009). Swimming movements of aquatic vertebrates differ considerably from the limbed locomotion of terrestrial vertebrates. Whereas fishes mainly use side-to-side flexion of the torso to shift in water, most extant tetrapods mainly use their limbs for propulsion, with trunk movements playing a subsidiary role in the potentiation of limb movements. In addition, legged-locomotion on land places unique demands related to weight-loading, postural, and limb control on uneven terrains.

We still do not know how the swimming CPG has been modified during evolution to sustain legged-locomotion. The transition may have been gradual, since amphibians and reptiles show oscillatory movements of the axial body that are tightly coupled to limb movements. Limb movements may have resulted from a reconfiguration of the swimming CPG at limb metamers, or they may have resulted from the addition of specialized modules controlling limb flexor–extensor muscles (Goulding, 2009). Some animal species can sustain swimming and walking at different developmental stages; for instance, amphibia shift from one locomotor mode to the other one from larval to adult metamorphosis (Combes et al., 2004). Also, as noted above, the zebrafish exhibits walking-like movements of the pectoral fins during slow swimming (Thorsen et al., 2004), indicating that some neural substrates for a walking mode are already present in teleosts (Goulding, 2009).

While most spinal cord is involved in the control of swimming in aquatic species (with a rostro-caudally traveling wave, phase-shifted in adjacent axial myotomes), CPGs at cervical and lumbar-sacral levels control the forelimbs and hindlimbs, respectively, of walking mammals. In particular, the isolated lumbar and sacral spinal cord is able to generate quasi-normal walking at the hindlimbs. According to one view, the CPG network for each limb may include multiple inter-connected modules controlling the movement of each joint, with a coupling of CPG activity across limb joints. The ability to generate rhythmic motor output is not evenly distributed in the lumbo-sacral cord, but there is a rostro-caudal excitability gradient (Deliagina et al., 1983; Cazalets and Bertrand, 2000; Kiehn, 2006). Indeed, isolated rostral segments (L1–L3 in rodents, L3–L5 in cats, D7–D10 in turtles) exhibit a stronger rhythmic drive than isolated caudal segments (L4–L6, L6–S1, and S1–S2, respectively). The stronger rhythmic drive of the rostral cord (which contains hip MNs) suggests that these segments act as leading oscillators, entraining more caudal and less excitable oscillators (perhaps those controlling knee and ankle joints). Motor bursts propagate rostrally and caudally from the lumbar region to farther segments (Falgairolle and Cazalets, 2007). In addition to autonomous signals generated within the spinal networks, sensory signals from joint, muscle, and skin receptors play a major role in shaping locomotion. In particular, sensory signals are involved in the onset and on-line adjustment of the locomotor rhythm, they can affect the amplitude and phase of the activity profiles in motor output, and their central effects are gated temporally with the result that reflexive contributions become appropriate to the specific phase of the step cycle (Pearson, 2000).

## Control Modules for Locomotion

Although the evidence reviewed above suggests the existence of modularly organized circuits in spinal CPGs, their exact functional structure remains unclear. Modularity might be organized at a segmental level, involving the control of single joints (in limbed-animals) or single axial metamers (in swimming animals). While this scheme appears compatible with the organization of swimming CPGs (as in the lamprey, Grillner, 2006), it appears unlikely in mammals. Thus, in both cats and humans, single joint movements are controlled by distinct spinal segments, because muscles involved in joint flexion and extension are innervated in several different segments even far apart. Alternatively, there could exist distinct flexor and extensor modules spanning multiple joints (or metamers), distinct from the commissural modules (including commissural interneurons) involved in left-right alternation. However, in both cats and humans, flexor and extensor muscles can be co-activated in a given phase of the gait cycle, and it is not evident whether this co-activation reflects shared or independent drives directed to flexors and extensors. Still another possibility is that there exist distinct rhythm-generation and pattern-generation modules (McCrea and Rybak, 2008). This reflects the idea of multi-layered organization of CPGs. Neurons in the rhythm-generating module would be two or more synapses upstream from MNs and project to pattern-generating neurons; the latter project monosynaptically to MNs. The main evidence for separate control of rhythm and pattern is provided by the observation that CPGs can maintain the period and phase of locomotor oscillations both during spontaneous deletions of MNs activity and during sensory stimulation affecting MNs activity (McCrea and Rybak, 2008).

## Development of Locomotion

Further cues about control modules are provided by developmental studies of locomotor patterns (Lacquaniti et al., 2012a). It has recently been shown that the primitive stepping patterns exhibited by human babies are retained and tuned, while new patterns are added during development (Dominici et al., 2011). Stepping can be elicited in newborns supported under the arms in an upright, slightly tilted forward posture, after contacting ground with the feet soles. Reflex stepping has been reported also in premature infants at 30+ weeks post-conception (Allen and Capute, 1986) and anencephalic newborns (Peiper, 1961). This is consistent with a predominant role of spinal and brainstem mechanisms, at a time when cerebral connections to the spinal cord are still immature. The neural patterns of muscle control have been studied by factorization of EMG activity into the basic components (Dominici et al., 2011). In human newborns, two patterns were sinusoidally modulated over the step cycle: one pattern was timed around the body support phase of stance, while the other one was timed during swing. Toddlers (∼1-year-old) at their first independent steps showed the same two patterns of the newborn, and 2 new patterns timed at touch-down and lift-off, probably contributing the shear forces necessary to decelerate and accelerate the body, respectively. In preschoolers (2–4-years), all four patterns showed transitional shapes: the older the child, the closer the waveform to the adult.

The development of adult gait from infant stepping depends on a progressive integration of supraspinal, intraspinal, and sensory control (Yang et al., 1998). In particular, the lack of muscle patterns around foot contact in the neonate might depend on immature sensory and/or descending modulation of stepping. Without sensory modulation (as in fictive locomotion), the spinal circuitry of animals also generates sinusoidal-like patterns (Falgairolle and Cazalets, 2007), similar to those observed in the human neonate. The addition of basic patterns in the first months of life implies a functional reorganization of inter-neuronal connectivity, the appearance of additional functional layers in the CPGs, and/or more powerful descending and sensory influences on CPGs.

Locomotor-like oscillatory activity can be recorded from the lumbar and sacral ventral roots of the isolated spinal cord of neonatal rats, bathed with dopamine plus NMDA or serotonin (Falgairolle and Cazalets, 2007). Factorization of the electroneurograms associated with this fictive walking showed two patterns essentially identical to those of human newborns (Dominici et al., 2011). Factorization of the EMG of adult rats, cats, macaques, and guinea fowls showed four patterns, closely resembling those found in human toddlers (Dominici et al., 2011). However, with development, the motor patterns may become tuned to the specific biomechanical requirements of each animal species. Thus, brief, pulsatile activations timed at the apex of limb oscillations is specific of human adult locomotion, perhaps in relation to our unique erect bipedal locomotion on extended legs and a heel-contact well ahead of the body (Dominici et al., 2011).

Also the topographical maturation of intraspinal connections in human babies is reminiscent of the organization observed in other animals. In rodents, turtles, and cats, a rostro-caudal excitability gradient has been described in the lumbo-sacral CPGs (Kiehn, 2006). Also human newborns exhibit a higher activation of lumbar vs. sacral segments (Ivanenko et al., 2013). With human development, the lumbar and sacral loci of activation become more dissociated with shorter activation times (Ivanenko et al., 2013), but the upper lumbar CPG may represent a major pacemaker also in human adults (Gerasimenko et al., 2010), whereas the sacral CPG could play a subordinate role for adaptation to specific foot-support interactions (Selionov et al., 2009). Overall, these behavioral results are consistent with the genetic and electrophysiological studies reviewed above which demonstrate that, despite the existence of species-specific features, there are several common principles in the neural organization and regulation of CPGs.

## Conclusion

The goal of identifying modules based on independence of function has been reached only in selected cases so far. In particular, experimental attempts to associate specific functions to individual modules of locomotor control mainly involved simple correlations between the temporal changes in biomechanical parameters and the parallel changes in muscle activity (Lacquaniti et al., 2012b). Computer simulations have also been used to correlate biomechanics and muscle activity (Neptune et al., 2009). The evolutionary developmental perspective may provide an alternative and fruitful approach by considering how developmental units match specific adaptive functions in different organisms (Kirschner and Gerhart, 1998; Schlosser and Wagner, 2004).

Although the evolutionary developmental approach is still in its infancy in the field of motor control, some general principles have already emerged. For example, the developmental addition of new premotor modules appears to underlie the postnatal acquisition and refinement of several different motor behaviors in vertebrates. We reviewed how new control patterns are added to the primitive ones during the development of human stepping (Dominici et al., 2011). Similar principles have been uncovered in the development of song. Thus, juvenile zebra finches initially produce babbling-like sub-songs, and start generating more mature songs with recognizable phrases around the seventh week after hatching (Aronov et al., 2008). This transition follows the formation of synaptic connections between the song premotor nucleus HVC and the motor nucleus RA, suggesting that more mature songs depend on the developmental addition of the HVC module to the premotor pathway (Aronov et al., 2008). Another example is provided by the development of murine vibrissal circuitry (Takatoh et al., 2013). Mice start employing rhythmic sweeping of their vibrissae for exploration around 2 weeks after birth. At about the same time, new sets of bilateral excitatory inputs are added to vibrissa facial MNs from neurons in the lateral paragigantocellularis nucleus (Takatoh et al., 2013). Moreover, descending axons from the motor cortex directly innervate these premotor neurons.

In general, modules can be conserved in their basic structural *bauplan*, but can lead to divergent morphologies and functions. Like a child with the Meccano^®^ or Lego^®^ parts, Nature has constructed a multitude of different forms and behaviors starting from a basic set of components over millions of years of evolution. Different animal species have gross morpho-functional differences, but they often use surprisingly similar organizational modules. A striking example is provided by the comparison of reaching strategies used by man and octopus, an invertebrate. Despite the evolutionary gap and morphological differences, humans, and octopuses evolved similar strategies to reach for a target (Sumbre et al., 2006). Thus, arm extension in octopus is controlled by basic muscle synergies involving the activation of all arm muscles (Sumbre et al., 2006), similar to the synergy control of human reaching (d’Avella and Lacquaniti, 2013). Moreover, octopus arms generate a quasi-articulated structure based on three dynamic joints with a tight temporal co-variance of joint motions (Sumbre et al., 2006). This kinematic invariance is closely reminiscent of the joint motion co-variance of human reaching movements (Soechting and Lacquaniti, 1981).

To make a simplistic metaphor, the flexible use and combination of similar basic components resulting in widely different behaviors could be equated to the different expression of similar genes resulting in widely different phenotypes and behaviors in different animal species. Novel evolutionary characteristics might have emerged from changes in expression of basic control patterns phylogenetically conserved, rather than the expression of totally new patterns. Here, we argued that features that are conserved across species might be modules that are recombined during evolution for the emergence of new phenotypes. However, an alternative possibility that currently we cannot rule out for complex organizations at systemic level, such as the motor patterns, is that their conservation is explained just as a result of convergent evolution, that is, they result from similar environmental pressure (natural selection).

## Conflict of Interest Statement

The authors declare that the research was conducted in the absence of any commercial or financial relationships that could be construed as a potential conflict of interest.
